# Prevalence of Bruxism in Athletes: A Systematic Review and Meta‐Analysis

**DOI:** 10.1111/joor.14039

**Published:** 2025-06-02

**Authors:** Alka Daby Nascimento de Sales, Renata de Albuquerque Cavalcanti Almeida, Anila Thais Lucena Barbosa, Jakelline Cipriano dos Santos Raposo, Jânio Luiz Correia Júnior, Beatriz de Araújo Gusmão, Ricardo Freitas Dias, Fabiana de Godoy

**Affiliations:** ^1^ Postgraduate Program in Hebiatrics ‐ Determinants of Health in Adolescents University of Pernambuco (UPE) Recife Pernambuco Brazil; ^2^ Department of Dentistry University of Pernambuco (UPE) Recife Pernambuco Brazil; ^3^ Federal Institute of Alagoas (IFAL) Maceió Alagoas Brazil

**Keywords:** athlete, athletic, bruxism, epidemiology, sleep bruxism, teeth grinding disorder

## Abstract

**Background:**

Bruxism is a behaviour that can be associated with negative health consequences, which may be exacerbated in athletes due to their exposure to anxiety situations during pre‐competition and competition periods.

**Objective:**

This study aimed to synthesise available scientific evidence investigating the prevalence of sleep and/or awake bruxism in athletes.

**Methods:**

This study followed PRISMA and PROSPERO (under number CRD42022301596). A literature search was conducted in 10 databases and grey literature. There were no restrictions on time or language, and observational studies demonstrating the prevalence of possible, probable, and/or definite sleep and/or awake bruxism in athletes and para‐athletes, including both athletes and para‐athletes from competitive categories, were included. No limitations were applied regarding the type of athlete, sport, age, or category (amateur or professional). The Joanna Briggs Institute Prevalence Critical Appraisal Tool was used to assess the risk of bias in the selected studies.

**Results:**

A total of 1214 studies were identified, and 23 studies were included in the review. There was a predominance of cross‐sectional studies (*n* = 16), with the number of athletes ranging from 20 to 370; the majority were male. Most studies have addressed bruxism generally without differentiating between awake and sleep bruxism (*n* = 14). The meta‐analysis estimated the overall prevalence of bruxism in athletes at 34% (95% CI = 0.26–0.42), being higher in the subgroup of athletes (0.36; 95% CI = 0.27–0.47) than in para‐athletes (0.27; 95% CI = 0.19–0.38).

**Conclusion:**

This study provided an estimate of the overall prevalence of bruxism in athletes and para‐athletes, although it was not possible to determine this prevalence by sex, age, category, and sports discipline because of the limited number of studies reporting this prevalence separately. Regarding bruxism assessment, clinical examinations and questionnaires were used, with the majority using self‐report questionnaires.

## Graphical abstract

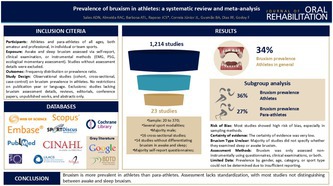

## 1 Introduction

Bruxism is a repetitive masticatory muscle activity characterised by teeth clenching or grinding and/or jaw immobilisation or thrusting. It is classified as sleep bruxism (SB) and awake bruxism (WB). In healthy individuals, it is not considered a disorder, but it can be a risk factor for negative consequences for oral health [1].

This behaviour can lead to significant sequelae in the stomatognathic system, such as dental wear, temporomandibular disorders, headaches, or fatigue of the masticatory muscles, as well as social complications such as compromised sleep quality [2, 3]. Sport can also be a risk factor for the occurrence of some oral diseases such as dental trauma, erosion, and periodontal diseases. Some of these conditions may not be linked to a specific sport, while trauma may be related to contact sports [4, 5].

Psychological factors, such as anxiety and stress, are associated with bruxism, regardless of age. The proposed pathophysiology is that individuals with higher levels of stress, anxiety, and neuroticism tend to release emotional tension, leading to SB and/or AB behaviours [6].

This behaviour can be associated with moderate to vigorous physical exercise [7] and is present in athletes [8, 9]. With the increasing specialisation in sports and the need to achieve even greater records, athletes are required to undergo more intense training to prepare them for maximum performance during competitions [10]. To achieve their goals, athletes' health must be in perfect condition [11].

Athletes are more predisposed to report anxiety than non‐athletes, either due to the high intensity of training or psychological pressure during competitions and increased muscular effort during training [12, 13]. Despite sports practice being related to a healthy lifestyle, oral diseases are frequently found in athletes. Within this context, bruxism and its consequences, such as tooth wear and fractures, can negatively impact well‐being, training, performance, and overall health [3, 10, 14].

On the other side, few meta‐analyses have examined bruxism prevalence; but none have focused on athletes. Studies report varying rates across populations: 46% in children with cerebral palsy [15]; in adults, awake bruxism prevalence is around 15.44% (99% CI = 10.81–20.72) [16]; among children, sleep bruxism ranges from 25.77% (95% CI = 22.16–29.38) to 31.16% (95% CI = 22.18–40.92) [17, 18]. Global estimates indicate 20.99% (95% CI = 18.69–23.50) for sleep bruxism and 23.29% (95% CI = 18.78–28.52) for awake bruxism [19]. While bruxism may impact physical performance, research on athletes is lacking, despite evidence that oral disorders can reduce performance by up to 21% [20]. Thus, the present study evaluated the prevalence of sleep bruxism and/or awake bruxism in athletes through a systematic review and meta‐analysis.

## 2 Methods

This systematic review and meta‐analysis study followed the recommendations of PRISMA [21] and PROSPERO [22] (CRD42022301596).

### 2.1 Eligibility Criteria

The research question followed the PECOS acronym strategy, as follows: population, athletes; exposure, bruxism; comparison, not applicable; outcome, prevalence of bruxism in athletes; and study types, observational studies.

#### 2.1.1 Types of Participants

The target population included athletes of both sexes, with no age limit, including both athletes and para‐athletes from competitive categories. All studies that defined their target population as athletes or individuals who trained and competed in sports or Olympic categories, including both amateur and professional athletes, in individual or team modalities were considered.

#### 2.1.2 Type of Exposure

Bruxism, both awake and sleep bruxism, is assessed through non‐instrumental tools (self‐reported and clinical examination) or instrumental approaches (electromyography, polysomnography, ecological momentary assessment) [1]. Studies that did not specify how bruxism was assessed were excluded from this review. Some authors did not use the term “bruxism” but rather “clenching and/or grinding.” For these studies, one of the authors, a specialist in the field, was consulted to determine whether the studies assessed bruxism following the international consensus.

#### 2.1.3 Types of Outcome Measures

Frequency distribution or prevalence ratio.

#### 2.1.4 Study Types

We exclusively included observational studies (cohort studies, cross‐sectional studies, case–control studies) that assessed the prevalence or frequency [23] of possible, probable, and/or definite sleep bruxism and/or wake bruxism in athletes and para‐athletes, with no limitation on the year of publication and language. Studies were excluded if they: (1) did not demonstrate how bruxism was assessed or did not answer the research question; (2) were review studies; (3) were editorials; (4) were conference publications; (5) were unpublished articles; (6) presented only the abstract.

### 2.2 Electronic Searches

The literature search was initially conducted on July 1, 2022, and updated on December 11, 2024. The search included the following electronic databases: Web of Science, Embase, PubMed, Virtual Health Library (VHL), Scopus, SPORTDiscus, CINAHL, LILACS (via VHL), Cochrane Library, and SciELO. Grey literature was searched using Google Scholar and the Brazilian Digital Library of Theses and Dissertations (BDTD). The searches in electronic databases were performed without language or year restrictions. The descriptors were searched in the MeSH and *DeCS/MeSH* dictionaries, combining terms using the boolean operators OR and AND (Data S1). The search was conducted by two researchers (AS and AT), independently and in pairs.

### 2.3 Selection Process

Two reviewers (AS and AT) independently screened the titles and abstracts of all retrieved references. We obtained the full‐text study reports for all citations that at least one review author considered potentially relevant. Two reviewers (AS and AT) independently screened the full‐text articles, identified studies for inclusion, and recorded reasons for excluding ineligible studies. Disagreements regarding study eligibility were resolved by a third author (JR). We identified and excluded duplicates by compiling multiple reports of the same study, with each study rather than each report being the unit of interest in the review. Reference management softwares, including EndNote Web and Rayyan, was used.

### 2.4 Data Collection Process

From each included study, two reviewers (AS and AT) independently extracted data, while a third reviewer (JR) ensured reliability and resolved any conflicts.

Primary data extraction covered study characteristics, such as population details, setting, sociodemographic characteristics, exposure, outcomes, and study design, all done in duplicate. We cross‐verified the primary study data for prevalence, resolving disagreements through discussion or consultation with a third reviewer.

The following information was extracted for each included study:

1. Background: period when the study occurred; type of publication (e.g., full‐text journal article, abstract, conference paper, thesis); study country or countries; author, year of publication; type of study.
2. Population and setting: population age and setting; participants (total number, mean age, proportion of each sex); type of athlete; sports category.
3. Methods: study design; description of the study.
4. Participants: total number; sociodemographic data.
5. Exposure: type of bruxism; bruxism assessment tool.
6. Outcomes: prevalence of bruxism in general; prevalence of sleep bruxism/awake bruxism according to age, sex, type of athlete, and sports category.
7. Other information: funding source, and conflicts of interest.

We used a pilot data collection form for the study characteristics and outcome data. If any information from the study was unclear or missing, we contacted the authors of the original papers for further details. Data were recorded on a data extraction form that summarised the key characteristics of the studies.

### 2.5 Assessment of Risk of Bias and Certainty of the Evidence

The quality assessment of the selected studies followed the checklist for prevalence studies from the Joanna Briggs Institute (JBI) Critical Appraisal tool for use in systematic reviews [24].

Two reviewers (AS and BG or JC and BG) independently assessed the risk of bias for each included study and scored each JBI question as yes, no, unclear, or not applicable. We have resolved any disagreements by discussion or by involving a third review author (JR or JC).

The certainty of the evidence was assessed for the pooled estimates using the Grading of Recommendations Assessment, Development, and Evaluation (GRADE) approach, which involved a systematic evaluation of the risk of bias, inconsistency, imprecision, indirect evidence, and other considerations [25].

Two reviewers (JC and JR) independently assessed the certainty of the evidence and scored each GRADE question as not serious, serious, or very serious. We have resolved any disagreements by discussion or by involving a third review author (RD).

### 2.6 Data Synthesis

The prevalence of bruxism in athletes was the main outcome and was expressed as a proportion of events of interest (PPs), and their respective 95% confidence intervals (95% CI) were calculated. A meta‐analysis of proportions with a random‐effect model was performed using the DerSimonian and Laird estimator [26]. Heterogeneity across studies was quantified using the Cochran's *Q* test, with statistical significance set at *p* < 0.1. Inconsistency was assessed through the Higgins' *I* ^2^ index, categorised as follows: ≤ 40%, low heterogeneity; 30% to 60%, moderate heterogeneity; > 50% to 90%, substantial heterogeneity; and > 75% to 100%, considerable heterogeneity. Furthermore, the Tau‐squared (*τ* ^2^) statistic was calculated to estimate between‐study variance. Significant heterogeneity was defined as *I* ^2^ > 50% and *τ* ^2^ > 1, coupled with statistical significance (*p* < 0.05) [27]. The Freeman‐Tukey Arcosene Double Transformation Method was used so that the data would follow an approximately normal distribution [28]. To stipulate the weight of the studies included in the analysis, the effect size of each study was weighted by the inverse‐variance method, calculating the estimate based on the inverse proportion of the study variance [28]. Confidence intervals of 95% (95% CI) were considered and calculated using the Clopper‐Pearson method. To investigate potential sources of significant heterogeneity, a subgroup analysis was conducted for categorical moderating variables by dividing the meta‐analysis groups according to the category of athletes reported in the included studies (e.g., athletes vs. para‐athletes) [29]. This approach was based on the hypothesis that athlete‐specific characteristics might influence the prevalence of bruxism.

#### 2.6.1 Sensitivity Analysis

A sensitivity analysis was performed to evaluate the robustness of the pooled effect size by systematically excluding studies classified as having a high risk of bias. This approach aimed to determine whether these studies significantly influenced the overall findings [30].

#### 2.6.2 Publication Bias

The publication bias was assessed using a funnel plot and confirmed using the Egger's test. Analyses and graphs were performed and plotted using the metapackage of statistical software Rstudio, version 4.1.3 (Rstudio Inc) [31, 32]. Graphic evaluation of the existence of publication bias was performed using the funnel graph, in addition to the Egger's test, to assess the existence of asymmetry in the funnel.

## 3 Results

### 3.1 Selection and Characteristics of the Studies

**FIGURE 1:**
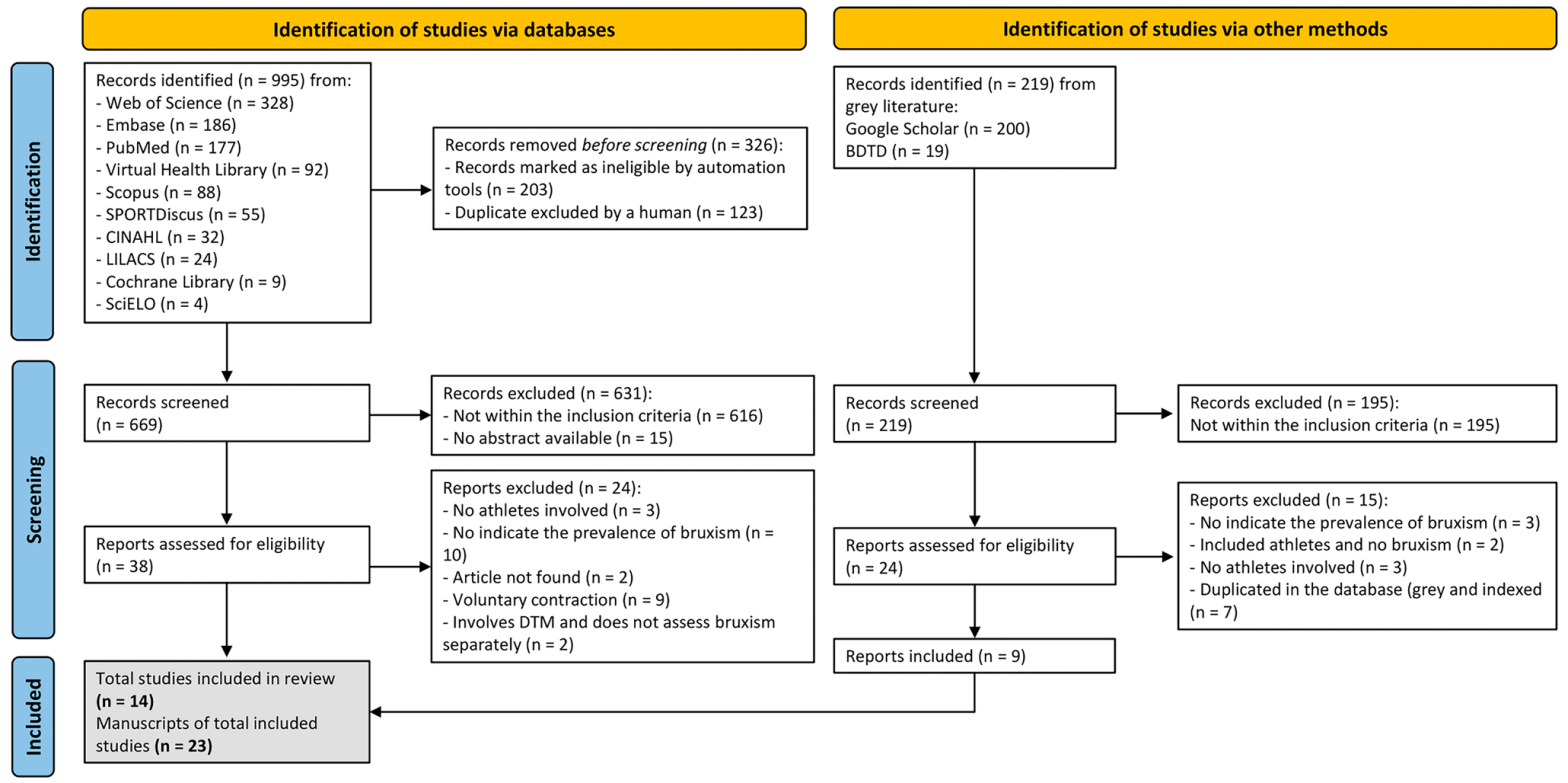
PRISMA flowchart.

A total of 1214 studies were identified. A flowchart detailing the study selection, inclusion, and exclusion process is presented below (Figure 1). The results' characteristics include data on the author, year of publication, study type, sample size, mean age, sex proportion, athlete type, bruxism type, sports category, bruxism assessment tool, and bruxism prevalence (Tables 1 and 2). Twenty‐three articles comprised systematic reviews, with studies ranging from 2002 to 2024. Among these, six were published between 2020 and 2022, nine were conducted in Brazil, three in Spain, one in Portugal, one in Thailand, one in Romania, and one in Poland.

**TABLE 1:** Description of included studies (*n* = 23).

Country	*n* (%)	Sample	*n* (%)
Brazil	11 (47.8)	Probabilistic	4 (17.4)
Italy	1 (4.3)	No probabilistic	3 (13)
Mexico	1 (4.3)	Not informed	16 (69.6%)
Poland	1 (4.3)	*Bruxism type*	*n (%)* ^a^
Portugal	2 (8.7)	Awake and Sleep bruxism (together)	5 (21.7)
Romania	1 (4.3)	Awake bruxism	2 (8.7)
Spain	3 (13)	Sleep bruxism	2 (8.7)
Thailand	1 (4.3)	Bruxism (type not informed)	14 (60.9)
Turkey	2 (8.7)	*Diagnostic tool* ^a^	*n (%)*
*Published date*	*n (%)*	Questionnaire	10 (43.5)
Before 2013^a^	2 (8.7)	Clinical inspection	5 (21.7)
Between/on 2013–2018	3 (13)	Questionnaire and clinical inspection	8 (34.8)
After 2018	18 (78.3)	*Modalities*	*n (%)*
*Study design*	*n (%)*	Olympic sports (various aggregated categories with or without contact sports)	10 (43.5)
Cross‐sectional	16 (69.6)	Contact sports	8 (34.8)
Case series	2 (8.7)	Rowing sports	2 (8.7)
Case–control	5 (21.7)	Not informed	3 (13)
*Type of athlete*	*n (%)*		
Para‐athletes	4 (17.4)		
Athletes	19 (82.6)		

^a^ Date before international consensus of bruxism.

**TABLE 2:** Description of the studies included (*n* = 23).

Study (year)	Participants	Type of athlete and modalities	Assessment tools	Outcome/measurements	Limitations (reported by authors)
Author: Babiuc et al. [33] Year: 2019 Design: cross‐sectional Funding: not reported Setting: sporting event in April 2018. (“Kayak‐Canoe, Romanian Cup for Seniors and Juniors”)	*n* = 34 (*M* = 58.8%; *F* = 41.2%) Age: 16–30 years Type of bruxism: bruxism (type not informed)	Athletes: high‐performance athletes (*n* = 34) Modalities: kayak and canoe	Assessment tool: questionnaire and clinical inspection Description: Questionnaire: each subject completed a questionnaire, giving details regarding genre, age, the type of sport he or she performed, the number of hours of training per day, personal history of dental pathology, and any perceived dental symptoms. Clinical inspection: A clinical examination was performed, identifying all the dental pathology associated with bruxism: tooth wear, decays, tooth fractures, alterations of the form of the alveolar crest	Bruxism: 100% (*n* = 34) Symmetrical bruxing symptoms: Kayak (*n* = 23): 82.6% Canoe right (*n* = 7): 14.3% Canoe left (*n* = 0): 0% Asymmetrical bruxing symptoms—right: Kayak (*n* = 23): 4.3% Canoe right (*n* = 7): 85.7% Canoe left (*n* = 0): 0% Asymmetrical bruxing symptoms—left: Kayak (*n* = 23): 13.1% Canoe right (*n* = 0): 0% Canoe left (*n* = 7): 100%	The number of athletes that were analysed was small
Author: Brancher et al. [34] Year: 2021 Design: cross‐sectional Funding: yes individual scholarships (FAPERJ–Fundação de Amparo à Pesquisa do Estado do Rio de Janeiro) Setting: regional selection Rio–Sul, organised by the Brazilian Par‐ Olympic Committee, April 12–14, 2019 in Curitiba, Paraná, Brazil	*n* = 271 (*M* = 66%; *F* = 34%) Age: 31.25 (±11.7) years Type of bruxism: bruxism (type not informed)	Athletes: para‐athletes (*n* = 271) Modalities: athletics: 147 Powerlifting: 61 Swimming: 63	Assessment tool: self‐reported questionnaire and clinical inspection Description: Questionnaire: The questionnaire addressed age, gender, ethnicity, and health data: body weight, height, use of isotonic or vitamin supplements, and sports practice. Additionally, questions about habits of clenching or grinding teeth were included. Clinical inspection: A team composed of dentists and note‐takers previously trained, and performed the para‐athletes' oral examination. Kappa value after calibration was higher than 0.90, indicating a high degree of data reproducibility. The presence of bruxism was confirmed by observation during clinical examination of signs and symptoms related to bruxism, such as dental wear facets and fractures of restorations; dental impressions on the cheek mucosa and tongue, based on a previous study	Bruxism: 37.9% (*n* = 89)	The dynamics imposed on para‐athletes on the day of competitions, reduce the anamnesis time
Author: Castilho et al. [35] Year: 2022 Design: cross‐sectional Funding: yes individual scholarships (FAPERJ–Fundação de Amparo à Pesquisa do Estado do Rio de Janeiro; PIBIC/CNPQ; CAPES) Setting: football team in Rio de Janeiro, Brazil	*n* = 34 (gender proportion not informed) Age: 22.35 (±4.72) years Type of bruxism: bruxism (type not informed)	Athletes: team players (*n* = 34) Modality: American football	Assessment tool: interview questionnaire and clinical inspection Description: Some data on dental history were obtained in the interview, using multiple choice questions. It assessed tooth sensitivity, diet, toothbrushing, toothpaste use, and intrinsic factors that could be related to dental erosion, teeth grinding, and dental tightening. Inter‐examiner reliability ranged from 0.80 (95% CI 0.80–0.95) to 1.00 and intra‐examiner reliability was Kappa =1.00	Bruxism: 29.4% (*n* = 10)	The sample size limitation
Author: Chantaramanee et al. [36] Year: 2016 Design: cross‐sectional Funding: not reported Setting: 2015 seasons of Phitsanulok Football Club, Thailand	*n* = 25 (gender proportion not informed) Age: 27.5 (±4.72) years Type of bruxism: bruxism (type not informed)	Athletes: professional soccer players (*n* = 25) Modality: soccer	Assessment tool: clinical inspection; interview, questionnaire Description: oral health status was examined by one dentist. Facial and oral trauma during training and performance were collected by questionnaire interview	Bruxism: 30% (*n* = 7)	The small number of sample
Author: de La Parte Serna; Fuente; Monticelli [37] Year: 2020 Design: case series Funding: not reported Setting: Centro de Medicina del Deporte del Gobierno de Aragón	*n* = 112 (*M* = 86.6%; *F* = 13.4%) Age: 24.57 (±4.82) years. Type of bruxism: bruxism (type not informed)	Athletes: elite or high‐performance athletes (*n* = 112) Modalities: not informed	Assessment tool: Clinical assessment; questionnaire Description: The dental examination was performed using the WHO standardised protocol and form for oral health assessment (Version 2013)	Bruxism: 59.8% (*n* = 67)	Not reported
Author: de La Parte et al. [8] Year: 2021 Design: case series Funding: not reported Setting: not informed	*n* = 186 (*M* = 80.6%; *F* = 19.4%) Age: 24.74 (±9.33) years. Type of bruxism: bruxism (type not informed)	Athletes: elite athletes (*n* = 186) Modalities: Individual: fencing, tennis, table tennis, athletics, rowing, canoeing, cycling, cross‐country skiing, alpine skiing, judo, triathlon, karate, trail running, paddle, badminton, orienteering, bicycle motocross, swimming, rhythmic gymnastics, climbing, and taekwondo Team: volleyball, basketball, ice hockey, handball, soccer, and water polo	Assessment tool: clinical assessment Description: Oral examinations were carried out by a single examiner who previously received instructions for performing oral examinations and completion of the registration forms. Intraexaminer concordance was assessed. A simple agreement percentage was used in the analysis (coincident diagnoses/total diagnoses × 100), observing an acceptable consistency with less than 3% error. An oral health assessment was performed through a dental examination using the WHO standardised protocol for adults (Version 2013)	Bruxism: 62.9% (*n* = 117)	No distinction was made between sexes, the nutritional intake of the participants was not analysed; there was no control group to compare results. The methodology of the present study should be repeated in a larger sample of athletes
Author: Gay‐Escoda et al. [38] Year: 2011 Design: cross‐sectional Funding: yes financial support from the Master of Oral Surgery and Implantology program‐healthcare agreement to the University of Barcelona, the Consorci Sanitari Integral and the Servei Català de la Salut of the Generalitat de Catalunya, Barcelona, Spain. Setting: Futbol Club Barcelona	*n* = 30 (gender proportion not informed) Age: 21(±1.6) years. Type of bruxism: bruxism (type not informed)	Athletes: professional soccer players (*n* = 30) Modality: soccer	Assessment tool: clinical examinations and interviews Description: the data were recorded by the same dentist to minimise inter‐examiner variability	Bruxism: 30% (*n* = 9)	Not reported
Author: Macêdo‐filho et al. [39] Year: 2019 Design: cross‐sectional Funding: no Setting: six jiu‐jitsu academies in the state of Paraíba, Brazil	*n* = 179 (*M* = 89.4%; *F* = 10.6%) Age: 16–40 years. Type of bruxism: bruxism (type not informed)	Athletes: beginners, intermediary, and advanced athletes (*n* = 179) Modalities: jiu‐jitsu	Assessment tool: clinical inspection Description: To aid in the diagnosis of centric bruxism (clenching the teeth), a physical examination was performed for the detection of an increment of the linea alba along the buccal mucosa and teeth marks on the edges of the tongue	Bruxism: 4.5% (*n* = 8)	Not reported
Author: Moreno [40] Year: 2019 Design: control–case Funding: no Setting: Sports Dentistry Department (MDD) of Clínica Universitária Egas Moniz and Lisboa Ginásio Clube and Sporting Clube de Portugal	*n* = 27 (*M* = 37%, *F* = 63%) Age: 19 (±1.97) years. Type of bruxism: awake bruxism	Athletes: competitive athletes (*n* = 27) Modality: olympic trampoline	Assessment tool: questionnaire Description: Bruxism Assessment Questionnaire (BAQ)	Bruxism: 41% (*n* = 11)	This study has some limitations, such as the correct use of BruxApp on smartphones. On smartphones with the Android operating system, there were some problems in using BruxApp due to the application's failure to alert users when they submitted their answers. As a result, responses were only obtained from 17 athletes. However, the author did not use this instrumental method to identify the prevalence of bruxism in the population; he only used it on the participants in the case group, who had already reported bruxism on the BAQ
Author: Queiroz et al. [41] Year: 2021 Design: cross‐sectional Funding: no Setting: 12 clubs affiliated to the Brazilian Rowing Confederation	*n* = 120 (*M* = 70%; *F* = 30%) Age: 24.16 (±5.74) years Type of bruxism: awake bruxism	Athletes: rowing athletes (*n* = 120) Modality: rowing	Assessment tool: questionnaire and clinical inspection Description: The questionnaire, which was written in Portuguese and answered anonymously, included 21 objective questions about the athletes' characteristics, clinical history, dental history, and questions related to sports practice, to assess some oral health parameters	Bruxism: 33.33% (*n* = 40)	Not reported
Author: Storrer et al. [42] Year: 2021 Design: case–control Funding: yes Universidade Positivo, CAPES – Coordenação de Aperfeiçoamento de Pessoal de Nível Superior and the Brazilian Paralympic Committee. Setting: Brazilian Paralympic Training Center located in São Paulo‐SP and Para‐Pan American Games that occurred in Curitiba‐PR	*n* = 370 (*M* = 67.3%; *F* = 32.7%) Age: Case group: 29.9 (±10.30) years Control group: 31.1 (±10.60) years Type of bruxism: bruxism (awake and sleep)	Athletes: para‐athlete (*n* = 370) Modalities: not informed	Assessment tool: questionnaire and clinical inspection Description: The oral examination was carried out by previously calibrated dentists in a room. The questionnaire also included information regarding the habit of clenching or grinding teeth. The presence of bruxism was therefore confirmed by observing signs and symptoms related to bruxism during clinical examination, such as dental wear facets and fractures of restorations, or dental impressions on the cheek mucosa and tongue and by the individual's self‐report	Bruxism: 38.4% (*n* = 142)	The limitation of this study was the absence of lesions at the time of the interview, so the collected responses were based only on self‐reports by the para‐athletes
Author: Zięba; Byś [35] Year: 2019 Design: cross‐sectional Funding: no Setting: Not informed	*n* = 88 (*M* = 53.4%; *F* = 46.6%) Age: 28 (±7) years Type of bruxism: awake and sleep bruxism	Athletes: climbers (*n* = 88) Modality: climbing	Assessment tool: questionnaire Description: The questions used to assess bruxism were constructed following the guidelines of Pintado et al., Lavigne et al., and Thorpy. Conscious bruxism was assessed by the following question: “Have you ever been aware of clenching or grinding your teeth during wakefulness in the past 6 months?” The answer “yes” qualified the subjects to the group suffering from awakening bruxism [13, 16]. The following questions were used to assess sleep bruxism: 1. “Are you aware, or has anyone heard you, grinding your teeth frequently during sleep?” 2. “Are you aware that your dentition is worn down more than it should be?” And 3. “Are you aware of any of the following symptoms upon awakening?”: (a) Sensation of fatigue, tightness, or soreness of your jaw upon awakening?; (b) Feeling that your teeth are clenched or that your mouth is sore upon awakening?; (c) Aching of your temples upon awakening?; (d) Difficulty in opening your mouth wide upon awakening?; (e) Feeling of tension in your jaw joint upon awakening and feeling as if you have to move your lower jaw to release it?; (f) Hearing or feeling a “click” in your jaw joint upon awakening that disappears afterwards? The subjects were assessed as suffering from night bruxism, if they answered “yes” to questions 1 and/or 2 and reported at least one symptom from question 3.	Bruxism: AB 51.1% (*n* = 45) SB 22.7% (20)	The small amount of scientific work in this area
Author: Bonotto et al. [43] Year: 2018 Design: cross‐sectional Funding: not reported Setting: Desterro de Florianópolis rugby team	*n* = 30 (*M* = 80%; *F* = 20%) Age: years Type of bruxism: bruxism (type not informed)	Athletes: semi‐professional players (*n* = 30) Modality: rugby	Assessment tool: questionnaire Description: validated Portuguese version of the Research Diagnostic Criteria for Temporomandibular Disorders (RDC/TMD)	Bruxism: 33.33% (*n* = 10)	
Author: Mello et al. [44] Year: 2002 Design: cross‐sectional Funding: unclear Setting: Federal University of São Paulo and Federal University of Pernambuco	*n* = 64 (*M* = 93.6%; *F* = 6.4%) Age: 26.3 (±5.9) years Type of bruxism: sleep bruxism	Athletes: para‐athletes (*n* = 64) Modalities: swimming, basketball, PC soccer, weightlifting, cycling, table tennis, athletics, judo and fencing	Assessment tool: questionnaire Description: sleep complaints questionnaire, which assesses, among other things, possible sleep disorders (sleepwalking, bruxism, apnea, asthma, tachycardia, headache, gastric reflux, snoring, cramps)	Bruxism: 11% (*n* = 7)	Not reported
Author: Silva et al. [45] Year: 2019 Design: cross‐sectional Funding: unclear Setting: Sleep Institute (Associação Fundo de Incentivo à Pesquisa – AFIP), São Paulo, Brazil	*n* = 146 (*M* = 59%; *F* = 41%) Age: 24.3 (±4.6) years Type of bruxism: sleep bruxism	Athletes: elite athletes (*n* = 146) Modalities: modern pentathlon, artistic gymnastics, canoeing, swimming, field and track, judo, beach volleyball, sailing	Assessment tool: questionnaire Description: a team of sleep specialists conducted the clinical evaluation using the UNIFESP Sleep Questionnaire developed and validated by the Sleep Institute/AFIP to evaluation of sleep disorders. During completion of the questionnaire, they asked the athlete about any sleep problems such as waking up tired, having insufficient sleep, insomnia, excessive daytime sleepiness, breathing complaints, restless legs, snoring, moving a lot during sleep, nightmares, light sleep, bruxism, talking in the sleep, and somnambulism	Bruxism: 1.2% (*n* = 3)	The limitations of the present study include the fact that athletes performed only one PSG, we performed no follow‐up of athletes to find out if there was an improvement in their sleep following the intervention, and that we did not analyses the results according to the athletes sport. The authors did not use the PSG to assess bruxism
Author: Weiler et al. [46] Year: 2013 Design: control–case Funding: not reported Setting: The Sport Clinic at the Federal University of Sao Paulo	*n* = 89 (*M* = 0%; *F* = 100%) Age: 10–18 years Type of bruxism: bruxism (type not informed)	Athletes: athletes of contact sports (*n* = 89) Modalities: basketball and handball	Assessment tool: questionnaire Description: Complete medical and dental histories were taken from the patients and a survey was used to assess the following signs and symptoms [14]: jaw pain when chewing, unusually frequent headaches (more than once a week and of unknown aetiology), stiffness/tiredness in the jaws, difficulty in opening the mouth wide, grinding teeth, and sounds at the TMJ. Each question was explained by the four previously calibrated examiners and could be answered with either “yes” or “no”	Bruxism: 33.33% (*n* = 30)	Not reported
Author: Aktaş et al. [47] Year: 2024 Design: control–case Funding: yes Atatürk University Scientific Research Projects Coordination Unit Setting: Universiade Winter Games hosted in Erzurum, Turkey	*n* = 91 (gender proportion not informed) Age: 12–16 years Type of bruxism: bruxism (type not informed)	Athletes: winter athletes (*n* = 91) Modalities: alpine skiing, snowboarding, biathlon, ski jumping, and ice hockey	Assessment tool: interview and clinical inspection Description: Data was collected in 2019 by one 1 experienced paediatric dentist. Calibration was conducted by comparing the previous oral examination results of the 15 selected athletes (examined by E.K.) to the diagnoses remade by 1 of the authors (F.S.) until the level of agreement had reached 90%. Individuals were questioned and examined for bad oral habits such as bruxism, teeth grinding, lip biting, cheek biting, and pencil biting	Bruxism: 33% (*n* = 23)^a^	Not reported
Author: Alemida et al. [48] Year: 2023 Design: cross‐sectional Funding: not reported Setting: not reported	*n* = 312 (*M* = 41%; *F* = 59%) Age: not informed years Type of bruxism: awake and sleep bruxism	Athletes: competitive athletes (*n* = 312) Modalities: powerlifting, strongman, crossfit, olympic weightlifting	Assessment tool: questionnaire Description: A questionnaire developed by the authors, consisting of 17 questions aimed at identifying athletes' performance levels and potential sequelae related to the stomatognathic system. The questionnaire included items on self‐perceived teeth clenching and the development of bruxism (teeth grinding during sleep)	Bruxism: AB = 31.4% (*n* = 98) SB = 13.5%% (*n* = 42)	Data collection was based on the athletes' self‐reported responses, which may introduce limitations regarding the accuracy and honesty of their statements
Author: Cardoso et al. [49] Year: 2023 Design: cross‐sectional Funding: yes Fundação para a Ciência e a Tecnologia (FCT) and European Union (EU) Setting: not informed	*n* = 116 (*M* = 61.2%; *F* = 38.7%) Age: 18.2 ± 4.1 years Type of bruxism: bruxism (type not informed)	Athletes: athletes of a high‐ and elite‐level (*n*) Modality: Swimming, athletics, tennis, canoeing, gymnastics, surfing, pentahlon, triathlon, shooting, sailing, equestrian	Assessment tool: clinical inspection Description: Intraoral health screening appointments were performed by an experienced clinical dentist. All procedures were minimally invasive and painless, aimed to make a gross assessment and for recognising abnormal conditions of dental relationships, teeth and periodontal health, and the presence of parafunctional activities (bruxism), current or past orthodontic treatment	Bruxism: 47% (*n* = 54)	Research among high‐level and elite athletes during training sessions is challenging given a substantial time commitment and potential interference to training, testing and competition schedules, they acknowledge some limitations. First, since oral health assessment were conducted using simple dental tools, without specialised sources including x‐ray, their oral health‐related data should be interpreted carefully given the potential for some oral diagnoses to be underestimated or not fully detected. Secondly, since their observations were not conducted under a dedicated clinical environment, we also acknowledge a limitation during the tooth decay assessment, particularly for the diagnosis of initial non‐cavitated “white” dental lesions where specific conditions are important for their detection (such as tooth dryness). Another limitation of our study is the fact that no comparison was made between high‐level and elite athletes, nor between sports, given the small number of athletes in some comparison groups, i.e., we did not have high‐level athletes in some sports and other sports had a small sample size (e.g., shooting, sailing and triathlon vs. swimming and athletics)
Author: Crincoli et al. [50] Year: 2022 Design: control–case Funding: no Setting: School of Dentistry of the University of Foggia, Italy	*n* = 100 (*M* = 100%; *F* = 0%) Age: 23.62 ± 5.7 years Type of bruxism: awake and sleep bruxism	Athletes: competitive athletes (contact sports) (*n* = 100) Modalities: soccer, rugby, American football, boxing, basketball	Assessment tool: questionnaire and clinical inspection Description: Bruxism was diagnosed through a non‐instrumental assessment based on self‐reported information (medical history, questionnaires) and clinical examination. Patients were asked whether they had the habit of grinding or clenching their teeth during the day or whether they had been reported to have this habit during sleep. Self‐reported assessment of sleep or awake bruxism continues to be the primary tool in bruxism research and clinical practice, so a positive self‐report was considered suggestive of possible bruxism	Bruxism: 20% (*n* = 20)	Regarding the recruitment of the CG, people referring to the hospital may complain of oral disturbances more frequently than the global population, so this could be a point of weakness of the present study. Another limitation lies in the decision of the Ethics Committee to allow only a clinical‐observational study, prohibiting I and II level radiological investigations. This clinical approach implies a less accurate diagnosis regarding the presence/absence of condylar alterations, such as arthritis or arthrosis
Author: Mendiburu‐Zavala et al. [51] Year: 2022 Design: cross‐sectional Funding: not informed Setting: service of the Unidad de Atención Integral de la Salud (UAIS) at the Universidad Autónoma de Yucatán (UADY), Mexico	*n* = 23 (not informed separately for the sample of athletes) Age: not informed separately for the sample of athletes Type of bruxism: awake and sleep bruxism	Athletes: high‐performance university athletes (*n* = 23) Modalities: not informed	Assessment tool: questionnaire Description: For evaluation, sleep bruxism was confirmed if the student responded positively to question 1 and/or 2 of the self‐report questionnaire (1. “Are you aware of clenching or grinding your teeth during sleep, either frequently or occasionally?” 2. “Has anyone told you that you grind your teeth while sleeping?”) or if they presented two or more of the following symptoms: teeth grinding accompanied by a characteristic sound that may even wake a bed partner; pain in the temporomandibular joint; pain in the masticatory and cervical muscles (i.e., myofascial pain); headache, particularly in the temporal region upon waking, which gradually subsides during the day; dental hypersensitivity; excessive tooth mobility sensation; poor sleep quality; and fatigue. For awake bruxism, confirmation was based on a positive response to question 1 and/or 2 of the self‐report questionnaire (1. “Do you clench your teeth while awake?” 2. “Do you rub your teeth together while awake?”) or the presence of the symptom “painful sensitivity of the masticatory muscles while awake.”	Bruxism^b^: 87% (*n* = 20) AB = 8.69% (*n* = 2) BS = 13.04% (*n* = 3) BV e BS = 65.21% (*n* = 15)	Not reported
Author: Üver, Yildiz [52] Year: 2023 Design: cross‐sectional Funding: not reported Setting: not informed	*n* = 338 (*M* = 71.3%; *F* = 28.6%) Age: 18–25 years Type of bruxism: bruxism (type not informed)	Athletes: individual and team sports athletes (*n* = 338) Modalities: badminton, basketball, football, handball, judo, martial arts (taekwondo/boxing/kickboxing/karate), swimming, skiing/snowboarding, volleyball, wrestling	Assessment tool: questionnaire Description: the Fonseca Anamnestic Index (FAI) was created in Brazilian Portuguese to evaluate the extent of TMD based on the indications and symptoms of the patient. FAI consists of 10 questions: ‘Yes’ (10 points), ‘No’ (0 points), and ‘Sometimes’ (5 points), to which the participant is asked to respond. The question about bruxism was: Do you have habits such as clenching or grinding your teeth?	Bruxism (clenching or grinding): 19.2% (*n* = 65)	Not reported
Author: Silva et al. [53] Year: 2022 Design: cross‐sectional Funding: unclear Setting: sports training center of the Federal University of Minas Gerais	*n* = 20 (*M* = 85%; *F* = 15%) Age: 33.65 ± 8.35 years Type of bruxism: bruxism (type not informed)	Athletes: para‐athletes (*n* = 20) Modalities: athletics, swimming and powerlifting	Assessment tool: questionnaire Description: Sleep complaints questionnaire. This questionnaire was developed as a clinical trial to sleep disturbances and complaints. It presents some complaints related to sleep such as “insufficient sleep”, “kicking legs”, “excessive daytime sleepiness”, “nightmares”, among others, and ask the participant to inform if the complaint occur or not, and if it occurs, the participant is asked to inform its frequency. There is no classification or score resulting from this questionnaire	Bruxism: 21% (*n* = 4)^a^	Some variables were cross‐sectionally analysed, and even for the variables with longer follow‐up, only associations were tested. Another limitation to highlight is the relatively small sample size. To estimate epidemiological data on sleep characteristics and injury/illness profile in Paralympic athletes, larger sample sizes are required. Yet, it is important to consider the difficulties in recruiting a large sample of Paralympic athletes to perform the sleep measurements used in this study. Furthermore, sleep and health problems data were collected during the post‐competition period, which may have increased the prevalence of sleep and health problems due to cumulative effects of the demand throughout the season

^a^ Informed by e‐mail by the authors.

^b^ Of the total sample of athletes (*n* = 23).

There was a predominance of cross‐sectional studies, with the number of athletes ranging from 20 to 370. Most participants were male, except for one article in which all participants were female. Three studies involved paralympic athletes. Regarding the type of bruxism, most of the articles did not specify the type, with only two studies assessing sleep bruxism (BS) and awake bruxism (BV) individually, and five studies assessing both bruxisms. The most prevalent instrument for assessment of bruxism was the self‐report questionnaire alone (43.5%), and the prevalence of bruxism in athletes varied from 4.5% to 100%, with the 100% value being found only in the study by Babiuc et al. [33].

### 3.2 Risk of Bias

**FIGURE 2:**
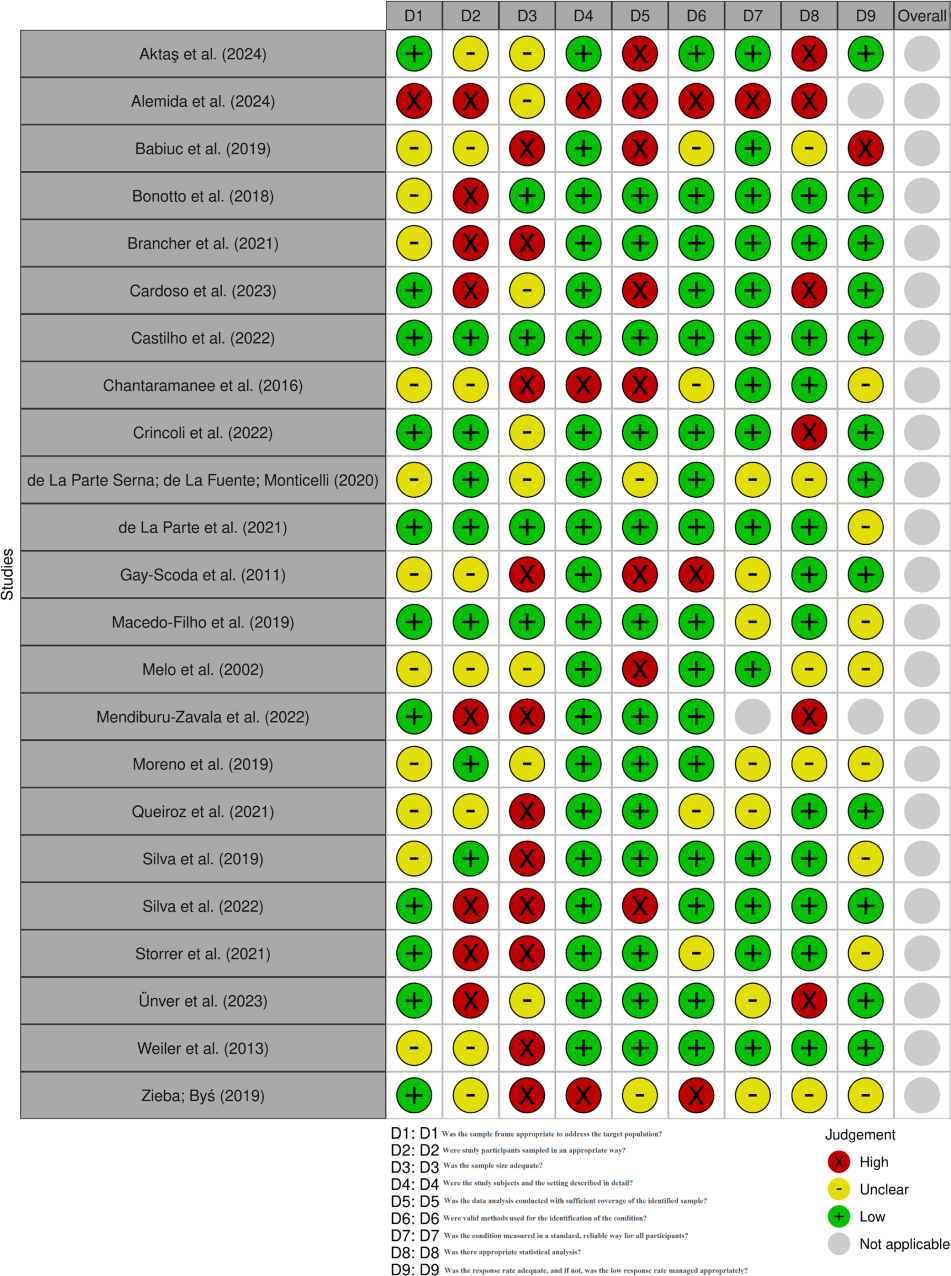
Risk of bias.

Regarding the risk of bias assessment, 11 studies demonstrated a high risk of bias for criterion 3 (Was the sample size adequate to address the target population?), eight studies for criterion 2 (Were participants in the study sampled appropriately?), and eight studies for criterion 5 (Was the data analysis conducted with sufficient coverage of the identified sample?). Additionally, three studies presented a high risk of bias for criterion 4 (Were the study participants and setting described in detail?) and criterion 6 (Were valid methods used to identify the condition?), while six studies were classified as high risk for criterion 8 (Was the statistical analysis appropriate?). Lastly, a single study exhibited a high risk of bias for criterion 1 (Was the sampling frame adequate to address the target population?), criterion 7 (Was the condition measured in a standard and reliable manner for all participants?), and criterion 9 (Adequacy of response rates and appropriate handling of low response rates). The following studies demonstrated a high risk of bias across multiple criteria: Aktaş et al. [47], Alemida et al. [48], Babiuc et al. [33], Brancher et al. [34], Cardoso et al. [49], Chantaramanee et al. [36], Gay‐Escoda et al. [38], Mendiburu‐Zavala et al. [51], Silva et al. [53], Storrer et al. [42], Ünver; Yildiz [52], Zięba; Byś [54] (Figure 2).

### 3.3 Summary of the Results

**FIGURE 3:**
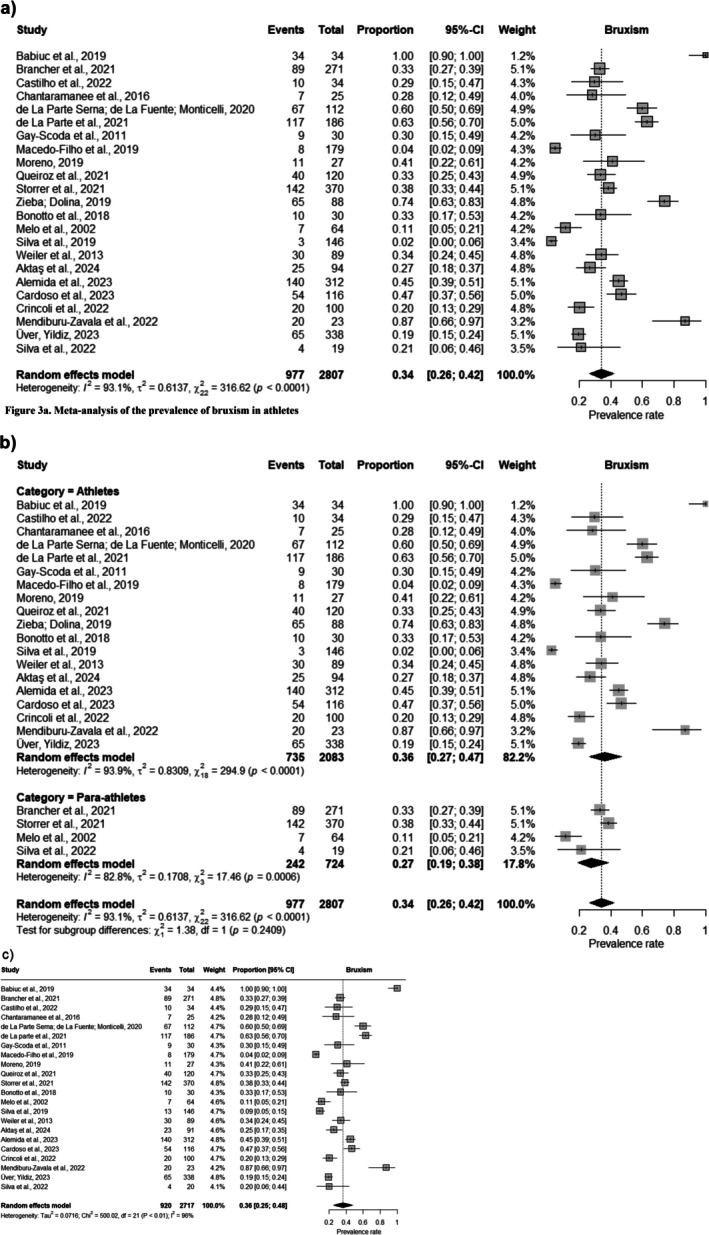
(a) Meta‐analysis of the prevalence of bruxism in athletes. (b) Meta‐analysis for the athlete and para‐athlete subgroups. (c) Meta‐analysis following sensitivity analysis.

The estimate of the overall prevalence of bruxism was based on including 2805 athletes in the analysis. The pooled prevalence of bruxism was calculated as 34% (95% CI = 0.27–0.47); *p* < 0.001. However, there was significant statistical heterogeneity between the studies (*I* ^2^ = 93.1%) among athletes (Figure 3). The application of the random‐effects model in the meta‐analysis was based on the significant statistical heterogeneity identified, considering the diverse sports categories and types of athletes included in the study. This model incorporates heterogeneity in the analysis, considering variations that may arise due to differences between studies.

To explore heterogeneity, subgroup analysis was performed, separating the studies according type of athlete (para‐athletes and athletes). The pooled prevalence estimate was 27% (95% CI = 0.19–0.38) for para‐athletes and 36.0% (95% CI = 0.27–0.47) for athletes (Figure 3b).

### 3.4 Sensitivity Analysis

A sensitivity analysis was performed to verify the influence of the study of Zieba; Byś [54] classified as having a high risk of bias on the observed summary effect about the prevalence of bruxism. After removing one study, the overall estimate of the meta‐analysis did not change significantly; the pooled prevalence was 0.3617 (95% CI = 0.2535–0.4771; *I* ^2^ = 96%) (Figure 3c).

### 3.5 Publication Bias

The graphical analysis of the funnel plot of prevalence of bruxism demonstrated the existence of studies with a higher standard error of the observed effect size for both sides of the summary estimate; that is, studies with smaller samples, and consequently greater standard error, were distributed symmetrically in the wider part of the funnel, for both sides of the summary estimate, suggesting the absence of publication bias. Likewise, the Egger's test confirmed the absence of publication bias (*p*‐value = 0.4216).

### 3.6 Certainty of Evidence

The certainty of evidence was very low according to the GRADE approach. The explanation is given by the risk of bias assessment of the included studies, unclear exclusion criteria, uncontrolled confounding factors, and large confidence intervals. The certainty of the evidence for the prevalence of bruxism in adolescents is very low. A decrease in the certainty of evidence occurred because of high heterogeneity (inconsistency) and wide CIs (imprecision) in the obtained estimates. Moreover, the existence of possible uncontrolled confounding factors may have distorted the estimates (Table 3).

**TABLE 3:** Evidence certainty assessment using the grading of recommendations assessment, development, and evaluation tool.

Certainty assessment							Summary of findings			
Number of studies	Study design	Risk of bias	Inconsistency	Indirectness	Imprecision	Other considerations	n° of events	n° of participants	Effect Proportion (95% CI)	Level of certainty of evidence
23	Non‐randomised studies	Serious^a^	Serious^b^	Serious^c^	Not serious^d^	None	975	2805	0.38 [0.28; 0.48]	⨁◯◯◯ very low

^a^ In general, item D3 (Was the sample size adequate?) presented a high risk of bias.

^b^ The effects of the studies are heterogeneous, as demonstrated by Chi^2^.

^c^ The eligibility criteria did not set the age limit, but only adolescents and adults were included.

^d^ The effect estimates differ little between the studies of each comparison.

## 4 Discussion

According to the meta‐analysis, the overall prevalence of bruxism among athletes was 34% (95% CI = 26.00–42.00), which is higher than the global prevalence of bruxism (both awake and sleep), reported as 22.22% (95% CI = 19.55–25.11) [19].

Physical exercise is believed to reduce stress, depression, and anxiety, thus improving the emotional well‐being of those engaged in physical activity [55]. However, sports may have the opposite effect, causing tension, stress, physical exertion, and pressure, especially during competitions, potentially contributing to an overload of masticatory muscles, predisposing individuals to bruxism [8, 23, 42, 54].

Several factors may influence this difference in the athlete group, and these factors are explained in the sub‐chapters below.

### 4.1 Bruxism Definition

According to International consensus about bruxism, the definition of bruxism is recommended in two categories: sleep bruxism, when this behaviour occurs during sleep, and awake bruxism, when this behaviour occurs during wakefulness [1]. However, most studies presented bruxism in general terms without distinguishing between awake bruxism and sleep bruxism, referring to both simply as “bruxism” [8, 34, 36, 38, 39, 41, 43, 44, 48, 50, 51, 52, 53, 55]. In cases where a questionnaire was used, it was sometimes possible to determine whether the study investigated awake or sleep bruxism [48]. However, most studies did not explicitly specify the type of bruxism being examined, nor did they clarify whether the assessment tools used were validated.

This lack of specificity is a crucial limitation in bruxism research, as awake and sleep bruxism are distinct behaviours with different underlying mechanisms. Sleep bruxism is characterised by rhythmic or non‐rhythmic muscle activity, whereas awake bruxism is defined by repetitive or sustained tooth contact and/or mandibular bracing or thrusting. These differences can influence the manifestation of clinical signs and the associated consequences [1].

### 4.2 Bruxism Assessment

All studies included in this review assessed bruxism using non‐instrumental methods, such as questionnaires and clinical inspections [8, 33, 34, 35, 36, 37, 38, 39, 40, 41, 42, 43, 44, 45, 50, 51, 52, 53, 54, 55]. Silva et al. [45] utilised polysomnography (PSG), but not as a tool for sleep bruxism assessment. However, prevalence rates obtained through PSG tend to be higher. For instance, Zieliński et al. [19] reported a sleep bruxism prevalence of 43.42% (95% CI = 16.65–74.68). Nevertheless, these results showed a wide confidence interval, indicating considerable variation likely due to differences in study population, diagnostic criteria, and/or methodological approaches.

Self‐reported measures and clinical inspections have important limitations that may lead to either underestimation or overestimation of bruxism prevalence. In the case of self‐reporting, this issue is particularly relevant for sleep bruxism, as it often relies on observations made by a bed partner who reports teeth grinding during the night [56]. For awake bruxism, accurate self‐reporting depends on the individual's awareness of the behaviour and their ability to recognise it [57].

Similarly, clinical assessments may not always detect bruxism, as not all individuals exhibit visible signs such as tooth wear; it depends on the severity of bruxism. This is especially true for awake bruxism, where tooth contact may be intermittent or minimal, reducing the likelihood of detectable wear [58]. Or dental wear may have occurred some time ago, and the athlete may no longer exhibit bruxism behaviour [59]. Additionally, factors such as the consumption of energy drinks, meal frequency, sugar intake, and the presence of reflux symptoms in athletes can contribute to dental wear. Research indicates that frequent consumption of sports and energy drinks, which are often high in sugar and acidity, is associated with dental erosion among athletes [36, 60]. Moreover, the acidity in these beverages can exacerbate acid reflux, leading to further enamel erosion due to the introduction of stomach acids into the oral cavity [61].

In 2023 was published the Standardised Tool for the Assessment of Bruxism (STAB) was published as a way to standardise the bruxism assessment and include several aspects of evaluating bruxism and its consequences; however, this tool is still in the validation process and refinement [62]. Only one of the studies included was published after 2023, but the authors did not define or classify bruxism [47] according to the STAB or the International Consensus.

### 4.3 Sample

Regarding the sample characteristics of the included studies, the male sex predominated in most works, except in the study by Weiler et al. [46], in which all participants were women. The sex ratios in the included studies ranged from 58.8% to 89.4% for males and from 10.6% to 100% for females. Perhaps, this can be justified by the fact that sports environments are still predominantly male. A recent systematic review by Paul et al. [63] demonstrated that male athletes are disproportionately favoured in sports medicine research across most sports, even in co‐ed disciplines. The underrepresentation of female athletes in the scientific literature has been attributed to factors such as greater availability of public and database data for male athletes, financial and promotional incentives favouring male sports, the predominance of male sports medicine clinicians and researchers, and persistent sex biases in sports culture. These inequalities in study populations may contribute to a limited understanding of conditions that could present differently across sexes. However, a recent meta‐analysis showed that sleep and awake bruxism are more prevalent in women, which may have underestimated bruxism in this population [19].

The age groups varied among adolescents, young adults, and adults, with the average age ranging from 10 to 42 years. The prevalence of sleep bruxism may be higher in children than in adults for men, and the prevalence of vigilance bruxism is higher in adults for both sexes. In this review, two studies by Weiler et al. [46] and Aktaş et al. [47] exclusively investigated adolescents, both with a prevalence of 33% for bruxism, making it impossible to conduct subgroup analysis and estimate the prevalence of bruxism by age group.

Another important factor was the sample size; this topic demonstrated a higher risk of bias in most of the studies. Only four studies specified that their samples were probabilistic [8, 35, 50, 51], but in two of them, the criteria for sample selection were not provided [8, 51]. Sample size can significantly influence statistical outcomes, and while this topic remains debated, smaller samples may impact estimates of means, medians, Pearson correlations, chi‐square tests, and *p*‐values related to frequency. This issue is exemplified by the study conducted by Babiuc et al. [33], which included a small sample of 34 participants without an appropriate sample size calculation. This methodological limitation may have introduced bias and compromised the generalisability of results.

### 4.4 Group of Athletes

The meta‐analysis results showed a slightly lower prevalence in the group of para‐athletes (27%; 95% CI = 19.00–38.00). Regarding studies involving Paralympic athletes, the study by Brancher et al. [34] conducted in Brazil, in Curitiba, encompassed weightlifting, athletics, and swimming categories, presenting a bruxism prevalence of 37.9%. The study by Storrer et al. [42], also conducted in Brazil, with high‐performance, medium‐performance, and regional‐performance Paralympic athletes, though not specifying the sports category, found a prevalence of 38.4%. In the study by Mello et al. [44] with Brazilian Paralympic athletes from various categories participating in the Sydney Paralympics, the prevalence of sleep bruxism was 11%. In the study by Silva et al. [45] that investigated para‐athletes in athletics, swimming, and powerlifting, the prevalence of bruxism was 21%.

The differences in prevalence among these four studies may have been caused by the discrepancy in samples, with Mello et al. [44] and Silva et al. [45] having the smallest sample size and using only a self‐report questionnaire, which may have resulted in underreported outcomes. Storrer et al. [42] used a clinical examination combined with a questionnaire, similar to the study by Brancher et al. [34]. Brancher et al. [34] considered bruxism, clenching, and grinding teeth differently, defining bruxism solely as a clinical sign. They also reported the overall prevalence of bruxism, whereas Mello et al. [44] reported only the prevalence of sleep bruxism. In the article by Silva et al. [45], the bruxism data is not presented separately, as requested by the authors. Originally, the sample consisted of 20 athletes, but for the bruxism variable, there was one loss, resulting in a total of 19 athletes.

Regarding studies involving elite or high‐performance athletes, the prevalence of bruxism appears to be higher, possibly due to increased pressure and elevated stress levels contributing to its onset. In a study by de La Parte Serna, de La Fuente, Monticelli [37] conducted in Spain, the bruxism rate among high‐performance athletes in team sports was 59.8%. Another study [8], also in Spain, examined elite athletes across both individual and team sports, reporting a prevalence of 67.6% in individual sports and 59.8% in team sports.

Two studies had small sample sizes, with 34 and 23 athletes, respectively, in the studies by Babiuc et al. [33] and Mendiburu‐Zavala et al. [51], which may have affected the reliability of their findings.

Conversely, a study by Silva et al. [45] conducted in São Paulo, Brazil, across various individual and team sports, reported a prevalence of sleep bruxism (SB) of just 1.2%. However, the overall bruxism prevalence was not provided, only the rate for sleep bruxism. Differences in study methodology, including variations in sample sizes, bruxism definitions, and classification criteria, may contribute to these discrepancies.

It was not possible to determine which individual or team sports had a higher or lower prevalence of bruxism, as the studies did not differentiate between specific sports and had varying sample sizes. Notably, in the study by Silva et al. [45], during the research week, athletes refrained from training or competition to prevent any interference with sleep evaluation, which may have contributed to the low prevalence reported. Additionally, only sleep bruxism (SB) was assessed, whereas awake bruxism, which is more prevalent among athletes [54], was not considered.

### 4.5 Sport Modalities

Several sports modalities were investigated in the included studies, making it impossible to conduct a subgroup analysis. These modalities encompassed both individual and team sports and will be described comparatively within similar categories whenever possible.

#### 4.5.1 Rowing Sports

The study by Queiroz et al. [41], involving rowers from São Paulo and Rio de Janeiro, Brazil, reported a bruxism rate of 33.3%. In contrast, the study by Babiuc et al. [33], conducted with high‐performance Romanian athletes in the canoe and kayak categories, identified differences in the symmetry of bruxism symptoms: kayaking presented 82.6% of symmetric symptoms, while canoeing showed 85.7% of symptoms on the right side.

This may be associated with the fact that kayaking involves muscular effort on both sides, and depending on the exerted force, the athlete may unconsciously activate the bilateral masticatory muscles. This is because they are seated, pulling the paddle alternately, thus presenting these symptoms uniformly. In contrast, canoeing has a dominant side, and the athlete paddles on a specific side, facilitating contraction in the active region [33].

These two studies showed heterogeneity in the assessment of bruxism, potentially introducing an assessment bias. In the study by Babiuc et al. [33], a clinical examination was conducted, but calibration information was not provided, and details of how the examination was performed were not reported. In addition, the study exhibited a high risk of bias in some criteria of the JBI protocol. Queiroz et al. [41] solely relied on the self‐report questionnaire, which could have led to underreporting, as participants may not always be aware of bruxism. Another distinction lies in the sample sizes, with the study by Queiroz et al. [41] having a sample size four times larger than that of Babiuc et al. [33].

#### 4.5.2 Contact Sports

In Castilho et al. [35], involving American football athletes in Rio de Janeiro, the prevalence of bruxism was 29.4%. Similarly, in the study by Weiler et al. [46], also conducted in Brazil, in São Paulo, with adolescent athletes in high‐contact sports such as basketball and handball, the prevalence was 33.3%. The same prevalence was found in Bonotto et al. [43], conducted in Florianópolis, Brazil, with semi‐professional rugby athletes.

Concerning studies involving professional soccer athletes, in Gay‐Escoda et al. [38], conducted in Spain, the prevalence of bruxism was 30%. Similarly, in Chantaramanee et al. [36], with professional Thai football athletes, the prevalence was the same. Other similarities were observed between the studies, such as the samples and the number of training days, which differed in the number of weekly training hours. In the study of Gay‐Escoda et al. [38], athletes trained 4 to 5 times, totaling 8 to 12 h weekly, while in Chantaramanee et al. [36], they trained between 3 and 5 h per day for 5 days.

There were significant differences in the assessment conducted by Chantaramanee et al. [36], who identified that 8% of participants reported using muscle relaxants, but it was not clear whether this was on the day of the survey or earlier, as this could have influenced the results. The authors used clinical inspection and a self‐report questionnaire for bruxism evaluation. It was also reported that all participants consumed energy drinks, and approximately 36.5% of their sample had an association between dental wear and these beverages. In the study by Gay‐Escoda et al. [38], it was not clear whether they used any questionnaires because they mentioned creating a standardised screening protocol and then stated that all athletes underwent clinical examination and evaluation of parafunctional habits. They considered bruxism as a parafunctional habit but did not describe the evaluation criteria.

In the study by Macêdo‐filho et al. [39], with Jiu‐Jitsu athletes in the beginner, intermediate, and advanced categories in Brazil, a bruxism prevalence of 4.5% was detected. Moreno [40] researched adolescent and young Portuguese athletes competing in trampoline jumping. The prevalence of awake bruxism (AB) was 41% in the resting phase, 44.4% in the training phase, and 55.6% in the competition phase. In the study of Zięba; Byś [54], involving climbers, the prevalence of AB was 51.1% and that of sleep bruxism (SB) was 22.7%, slightly higher in females. The difference between these three studies may have been attributed not only to discrepancies in sample size, as the sample in the study by Macêdo‐Filho et al. [39] was almost six times larger than that of Moreno [40], but also to differences in the types of sporting practices analysed. Variations in sport modality can influence the outcomes, given the distinct physical, psychological, and social demands associated with different sports.

Crincoli et al. [50] investigated athletes from soccer, rugby, American football, boxing, and basketball, reporting a bruxism prevalence of 20%. Similarly, Ünver; Yildiz [52] found a prevalence of 19.2% in athletes from various sports, primarily contact sports. In contrast, most studies focusing on Olympic sports reported higher bruxism prevalence rates, ranging from 33% to 41%. When assessed separately, awake bruxism showed a higher prevalence (31.4%–51.1%) compared to sleep bruxism (1.2%–22.7%).

Despite similar prevalences among studies involving contact sports, some differences can be noted. A discrepancy between the studies by Weiler et al. [46] and Castilho et al. [35] lies in the sample size, with the former having a three‐times larger number of participants than the latter. Another disparity is related to the instrument used for bruxism assessment; the study by Weiler et al. [46] and Bonotto et al. [43] used only the questionnaire, whereas Castilho et al. [35] employed clinical examination combined with a self‐report questionnaire, with previously calibrated examiners, and all three studies showed a low risk of bias according to the JBI protocol criteria.

Understanding the influence of different sports on bruxism prevalence is challenging, particularly in studies that include multiple sports. However, it can be suggested that individual and team sports present distinct sources of stress and anxiety, which may impact bruxism prevalence. For example, in Olympic sports within individual categories, performance pressure is concentrated on the athlete, especially during competitions, where the focus is solely on their individual results. In contrast, team sports typically distribute pressure across the team, except in specific high‐stakes situations, such as taking a penalty kick, where an individual may experience heightened stress [64].

While external pressure in team sports may be less intense, they may still experience significant stress and anxiety related to their role within the team. If anxiety and stress are to be considered as factors influencing the prevalence of bruxism in athletes, it is essential to also evaluate the covariate of sex. Females may be more likely to report anxiety, yet the majority of participants in this review were male, which could influence the findings [64]. To better understand these differences, further research evaluating bruxism prevalence by sport category is needed.

### 4.6 Risk of Bias and Limitations

Regarding the risk of bias, the criterion that consistently showed a high risk of bias in most studies was related to sample characteristics. There was also a considerable disparity in the instrument tools used for bruxism, and all these factors may have influenced the prevalence results. The meta‐analysis revealed the presence of heterogeneity, stemming from studies with small samples and variability between studies. This variability may result from different instrument approaches, disparate samples, and diverse sports modalities. It is suggested to conduct studies with larger samples, distinguish between sports categories, and utilise PSG and EMG for bruxism assessment to obtain more reliable results.

This review systematised knowledge about the prevalence of bruxism in athletes and para‐athletes. However, most of the studies included presented a high risk of bias in various domains of the JBI, especially regarding the sample, but another point to consider is the bruxism studied and the timing of data collection. In Moreno [40], data on awake bruxism were collected at three different moments: before, during training, and 1 day before competitions. This is in contrast to Macêdo‐Filho et al. [39], who did not clarify the type of bruxism investigated and did not explicitly mention the timing of data collection.

## 5 Conclusion

This research enabled the estimation of the overall prevalence of bruxism in athletes and para‐athletes, although it was not possible to assess the prevalence of bruxism by sex, age, category, and sports discipline because of the lack of reported data in the included studies. The instruments used for bruxism assessment included clinical examinations and questionnaires, especially self‐reports.

## Conflicts of Interest

The authors declare no conflicts of interest.

## Peer Review

The peer review history for this article is available at https://www.webofscience.com/api/gateway/wos/peer‐review/10.1111/joor.14039.

## Supporting information


Data S1.

## Data Availability

Data available on request from the authors.
