# Supplementary material for: Prevalence of Bruxism in Athletes: A Systematic Review and Meta‐Analysis
Source: J Oral Rehabil. 2025 Jun 2;52(9):1518–43. doi: 10.1111/joor.14039 (PMC12408946; doi:10.1111/joor.14039)
Supplement: Supplementary file 1 — Data S1. [file JOOR-52-1518-s001.docx]

**Database search strategies**

***PubMed*** ***database***

#1 Athletes OR Athlete OR "Professional Athletes" OR "Professional Athlete" OR "Elite Athletes" OR "Elite Athlete" OR Sports OR Sport OR Athletics OR Athletic

#2 Bruxism OR "Sleep bruxism" OR "Awake bruxism" OR bruxomania OR clenching OR "teeth grinding" OR "dental grinding" OR "teeth clenching" OR "tooth clenching" OR "tooth grinding"

#3 #1 AND #2

***BVS and LILACS databases***

#1 Athletes OR Athlete OR “Professional Athletes” OR “Professional Athlete” OR “Elite Athletes” OR “Elite Athlete” OR Sports OR Sport OR Athletics OR Athletic

#2 Bruxism OR “Sleep bruxism” OR “Awake bruxism” OR bruxomania OR clenching OR “teeth grinding” OR “dental grinding” OR “teeth clenching” OR “tooth clenching” OR “tooth grinding”

#3 Atletas OR atletas profesionales OR atletas de elite

#4 bruxismo OR bruxismo nocturno OR bruxismo del sueño OR bruxismo de vigilia

#5 Atletas OR atletas profissionais OR atletas de elite

#6 Bruxismo OR bruxismo do sono OR bruxismo noturno OR bruxismo de vigília

#7 #1 AND #2

#8 #3 AND #4

#9 #5 AND #6

#10 #7 AND #8 AND #9

***CINAHL database***

#1 Athletes OR Athlete OR “Professional Athletes” OR “Professional Athlete” OR “Elite Athletes” OR “Elite Athlete” OR Sports OR Sport OR Athletics OR Athletic

#2 Bruxism OR “Sleep bruxism” OR “Awake bruxism” OR bruxomania OR clenching OR “teeth grinding” OR “dental grinding” OR “teeth clenching” OR “tooth clenching” OR “tooth grinding”)

#3 #1 AND #2

***Cochrane database***

#1 Athletes OR Athlete OR “Professional Athletes” OR “Professional Athlete” OR “Elite Athletes” OR “Elite Athlete” OR Sports OR Sport OR Athletics OR Athletic

#2 Bruxism OR “Sleep bruxism” OR “Awake bruxism” OR bruxomania OR clenching OR “teeth grinding” OR “dental grinding” OR “teeth clenching” OR “tooth clenching” OR “tooth grinding”

#3 #1 AND #2

***SciELO database***

#1 Athletes OR Athlete OR Professional Athletes OR Professional Athlete OR Elite Athletes OR Elite Athlete OR Sports OR Sport OR Athletics OR Athletic

#2 Bruxism OR Sleep bruxism OR Awake bruxism OR bruxomania OR clenching OR teeth grinding OR dental grinding OR teeth clenching OR tooth clenching OR tooth grinding

#3 #1 AND #2

***Sport Discus database***

#1 Athletes OR Athlete OR “Professional Athletes” OR “Professional Athlete” OR “Elite Athletes” OR “Elite Athlete” OR Sports OR Sport OR Athletics OR Athletic

#2 Bruxism OR “Sleep bruxism” OR “Awake bruxism” OR bruxomania OR clenching OR “teeth grinding” OR “dental grinding” OR “teeth clenching” OR “tooth clenching” OR “tooth grinding”

#3 #1 AND #2

***Scopus database***

#1 athletes OR athlete OR "Professional Athletes" OR "Professional Athlete" OR "Elite Athletes" OR "Elite Athlete" OR sports OR sport OR athletics OR athletic

#2 bruxism OR "Sleep bruxism" OR "Awake bruxism" OR bruxomania OR clenching OR "teeth grinding" OR "dental grinding" OR "teeth clenching" OR "tooth clenching" OR "tooth grinding"

#3 #1 AND #2

***Web of Science***

#1 Athletes OR Athlete OR “Professional Athletes” OR “Professional Athlete” OR “Elite Athletes” OR “Elite Athlete” OR Sports OR Sport OR Athletics OR Athletic

#2 Bruxism OR “Sleep bruxism” OR “Awake bruxism” OR bruxomania OR clenching OR “teeth grinding” OR “dental grinding” OR “teeth clenching” OR “tooth clenching” OR “tooth grinding”

#3 #1 AND #2

***Embase database***

('athletes'/exp OR athletes OR 'athlete'/exp OR athlete OR 'professional athletes'/exp OR 'professional athletes' OR 'professional athlete'/exp OR 'professional athlete' OR 'elite athletes'/exp OR 'elite athletes' OR 'elite athlete'/exp OR 'elite athlete' OR 'sports'/exp OR sports OR 'sport'/exp OR sport OR 'athletics'/exp OR athletics OR athletic) AND ('bruxism'/exp OR bruxism OR 'sleep bruxism'/exp OR 'sleep bruxism' OR 'awake bruxism'/exp OR 'awake bruxism' OR bruxomania OR clenching OR 'teeth grinding'/exp OR 'teeth grinding' OR 'dental grinding'/exp OR 'dental grinding' OR 'teeth clenching'/exp OR 'teeth clenching' OR 'tooth clenching'/exp OR 'tooth clenching' OR 'tooth grinding'/exp OR 'tooth grinding')

#3 #1 AND #2

***Google Scholar database***

(Athletes OR Athlete OR Professional Athletes OR Professional Athlete OR Elite Athletes OR Elite Athlete OR Sports OR Sport OR Athletics OR Athletic) AND (Bruxism OR Sleep bruxism OR Awake bruxism OR bruxomania OR clenching OR teeth grinding OR dental)

#3 #1 AND #2

***BDTD database***

(atletas OR atleta OR esportistas) AND (bruxismo OR bruxismo do sono OR bruxismo de vigília))**"**

#3 #1 AND #2
